# Gut microbiota changes in children with autism spectrum disorder: a systematic review

**DOI:** 10.1186/s13099-020-0346-1

**Published:** 2020-02-03

**Authors:** Lucius Kang Hua Ho, Valerie Jia Wei Tong, Nicholas Syn, Niranjan Nagarajan, Elizabeth Huiwen Tham, Stacey K. Tay, Shefaly Shorey, Paul Anantharajah Tambyah, Evelyn Chung Ning Law

**Affiliations:** 1 Yong Loo Lin School of Medicine, National University of Singapore, Singapore, Singapore; 2 Genome Institute of Singapore (GIS), Singapore, Singapore; 3 Singapore Institute for Clinical Sciences (SICS), Agency for Science, Technology and Research (A*STAR), Singapore, Singapore; 4 Department of Paediatrics, Yong Loo Lin School of Medicine, National University of Singapore, Singapore, Singapore; 5 Khoo Teck Puat-National University Children’s Medical Institute, National University Health System, Singapore, Singapore; 6 Alice Lee Centre for Nursing Studies, Yong Loo Lin School of Medicine, National University of Singapore, Singapore, Singapore; 7 Division of Infectious Diseases, University Medicine Cluster, National University Health System, Singapore, Singapore; 8 Department of Medicine, Yong Loo Lin School of Medicine, National University of Singapore, Singapore, Singapore

**Keywords:** Microbiome, Microbiota, Dysbiosis, Systematic review, Autism spectrum disorder

## Abstract

**Background:**

As more animal studies start to disentangle pathways linking the gut microbial ecosystem and neurobehavioral traits, human studies have grown rapidly. Many have since investigated the bidirectional communication between the gastrointestinal tract and the central nervous system, specifically on the effects of microbial composition on the brain and development.

**Methods:**

Our review at the initial stage aimed to evaluate literature on gut microbial alterations in pediatric neurobehavioral conditions. We searched five literature databases (Embase, PubMed, PsychInfo, Scopus, and Medline) and found 4489 published work. As the mechanisms linking gut microbiota to these conditions are divergent, the scope of this review was narrowed to focus on describing gut dysbiosis in children with autism spectrum disorder (ASD).

**Results:**

Among the final 26 articles, there was a lack of consistency in the reported gut microbiome changes across ASD studies, except for distinguishable patterns, within limits, for *Prevotella*, Firmicutes at the phylum level, Clostridiales clusters including *Clostridium perfringens*, and *Bifidobacterium* species.

**Conclusions:**

These results were inadequate to confirm a global microbiome change in children with ASD and causality could not be inferred to explain the etiology of the behaviors associated with ASD. Mechanistic studies are needed to elucidate the specific role of the gut microbiome in the pathogenesis of ASD.

## Background

Autism spectrum disorder (ASD) refers to a developmental and neurobehavioral condition characterized by deficits in social communication and social interaction across multiple contexts with restricted, repetitive patterns of behaviour, interests, or activities [1]. Recent data suggest that as many as 1 in 59 children are diagnosed with ASD, although other reports not using parental report and school age children generally show a prevalence of 1% globally, with little regional variations in developed countries within North America, Western Europe, Central Latin America, and Asia Pacific [2–7].

There is no single known cause for all ASD-related behaviors. Current research alludes to multifactorial etiologies including genetic risk factors, de novo mutations, gene-environment interactions, and environmental factors such as in utero exposures and perinatal events [2, 8]. Due to reports suggesting that children with ASD have increased prevalence of gastrointestinal symptoms including constipation, diarrhea, and abdominal discomfort, researchers have started to examine the differences in gut microbiome composition in these children [9–12].

Longitudinal studies on adults with ASD indicate that 37 to 59% have poor outcomes [13]. The average lifetime cost of supporting an individual with ASD is estimated to be at least USD$1.4 million in the United States and £0.92 million in the United Kingdom [14]. When a child has concurrent intellectual disability, this cost rises to USD$2.4 million and £1.5 million, respectively [14]. While autism-specific behavioral therapies have strong data supporting outcome improvement, there has not been reliable evidence on the effectiveness of environmental modifications including diet, antifungals, fecal microbiota transplants, heavy metal chelation, and vaccine avoidance. The intention of this review is not to discuss potential ways for intervention through gut microbiome modulation. Rather, it is to take a closer look at whether the plethora of literature published provides consistent evidence on features of gut microbiome alterations associated with ASD and to establish the strength of evidence.

### A new wave of interest in gut microbiome and autism spectrum disorder

Human studies have shown that children exposed to maternal inflammation during pregnancy have an increased risk for ASD, yet the mechanisms for this are poorly understood [15–17]. Since then, promising results from a number of landmark animal studies have revived considerable interest in linkages between ASD and the gut microbiome [18–21]. These animal studies have provided new evidence on mechanisms by which inflammation and gut microbiota influence neurobehaviors. For instance, pregnant mice with intestinal bacteria that induced activation of the maternal immune system, termed maternal immune activation (MIA), produced offspring with impaired sociability and repetitive marble burying behaviours [19]. These MIA-associated behaviours were reminiscent of ASD symptoms in humans. Furthermore, cortical patches dominantly localized in the primary somatosensory cortex were affected by MIA and were closely associated with these behavioral abnormalities [18].

Animal studies have also shown that changes in microbiota lead to changes in behaviors. Raising animals in the absence of microbial colonization, also called gnotobiotic environment, resulted in abnormalities in a variety of complex behaviours. For example, germ-free mice tended to exhibit decreased sociability and less propensity to interact with unfamiliar partners [22]. These same mice were found to have abnormalities in brain gene expression, display changes in their hypothalamic–pituitary–adrenal axis, and demonstrate adult hippocampal neurogenesis [22, 23]. Reintroduction of bacterial strains or restoration of gut microbial ecology in mice resulted in normalization of social behaviours. In one study, treatment with the gut bacterium *Lactobacillus reuteri* (*L. reuteri*) alone sufficiently reversed ASD-like symptoms in mice [21]. Alteration of the postnatal gut microbiota by early life treatment with the human gut bacterium *Bacteroides fragilis* (*B. fragilis*) also sufficiently ameliorated deficits in communicative and stereotypic burying behaviour in mice offspring exposed to MIA. A recent study showed that postnatal colonization with human “infant-type” *Bifidobacterium* species showed improved behaviours for gnotobiotic mice [24]. Together, these animal studies have hastened interest in human studies comparing gut microbiota between individuals with and without ASD.

### The human gut microbiota

The human gut microbiota contains a complex and dynamic population of microorganisms, which are believed to exert a broad effect on the host. Firmicutes and Bacteroidetes are two major microbial phyla in the gut. Both phyla are susceptible to alterations due to factors such as age, genetics, diet, environment, and infection and have roles related to immune dysregulation (e.g. lupus systemic erythematosus), systemic diseases (e.g. metabolic syndrome), and neurological disorders (e.g. Parkinson’s disease) [25].

The Firmicutes/Bacteroidetes ratio has been shown to change with age, with a ratio of approximately 0.4 in infants and as high as 10.9 in adults [26]. Among infants, there is also variability in the relative abundance of Firmicutes and Bacteroidetes. The most recent research demonstrates that clusters of infants with similar abundances of Firmicutes (i.e. the family *Ruminococcaceae*) and Bacteroidetes are associated with distinct cognitive and language profiles [27]. Studies on microbiome composition and ASD appear to suggest a trend of increased Firmicutes/Bacteroidetes ratio and reduced *Bacteroides* in the ASD groups compared to controls, leading prior reviews on this topic to support a role for the microbiome as an interface between environmental and genetic risk factors that are associated with ASD [28, 29].

However, there has not been a comprehensive review that systematically (1) evaluates the dysbiosis described in children with ASD based on bacterial taxonomy from phylum to species, (2) investigates whether results of dysbiosis are congruous in all cases, and (3) summarises both positive and negative findings down to species in all the studies captured. As such, our review aims to provide a detailed dissection of the current literature on gut microbiota and ASD.

To better understand this review, it is important to clarify that Autistic Disorder (AD) and Pervasive Developmental Disorder, not otherwise specified (PDD-NOS) are now both under the umbrella diagnosis of ASD in the Diagnostic and Statistical Manual for Mental Disorders, Fifth Edition, better known as DSM-5 (1). Studies published before DSM-5 with the diagnoses of AD and PDD-NOS are reported as ASD severe symptoms (severe) and ASD mild symptoms (mild), respectively, in this review to keep consistent with the current classifications.

## Methods

### Identification of studies

**Fig. 1:**
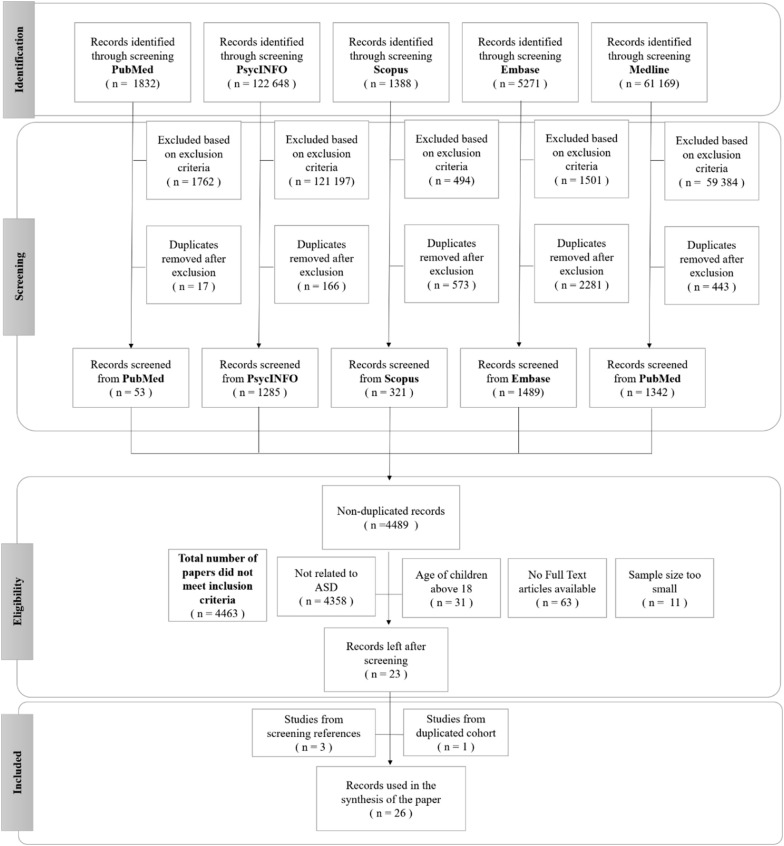
PRISMA flow diagram

A Preferred Reporting Items for Systematic Reviews and Meta-analyses (PRISMA) flow diagram of the study process is provided in Fig. 1. We conducted a systematic search of five literature databases to identify studies showing gut dysbiosis in neurological disorders. The databases searched were Embase, Medline, PsycINFO, PubMed and Scopus. All databases were searched in three waves, September 2017, August 2018, and April 2019, using the search criteria listed in Additional file 1: Table S1. The collections of papers were reviewed and duplicates were eliminated both electronically and manually. Articles were then screened based on titles and abstracts for eligibility.

The inclusion criteria were studies pertaining to (1) children under the age of 18 years of age with clinically diagnosed ASD, (2) more than 10 enrolled participants, (3) gut microbiota with descriptive and inferential statistics, and (4) full text peer-viewed articles. Exclusion criteria included studies with non-human subjects, single case reports or reviews, participants with genetic disorders that were associated with a high prevalence of ASD (e.g. Rett syndrome), concomitant condition of major diseases of the intestinal tract, as well as publication date earlier than January 2000 or after May 2019. We used 10 as the cutoff for the number of participants in our inclusion criteria. This parameter was chosen to ensure sufficient heterogeneity in the sample and to reduce the possibility of Type II errors.

Assessment of 23 articles for final inclusion was based on full text by authors L.H. and V.T. Disagreements on nine papers were resolved and arbitrated by authors E.C.L and N.S., and consensus was reached in all cases. Subsequently, the reference sections of all eligible articles were reviewed by E.C.L. and an additional three studies were found, which resulted in 26 articles.

### Data extraction

Data reported from each study were manually extracted from the full text articles to a database including: (1) study location, (2) study sample size for both case subjects and controls, (3) study type (longitudinal, cross-sectional, prospective, retrospective, randomized), (4) participant age range, (5) diagnostic criteria or assessment used, (6) molecular and microbiology methods, (7) interventions used, and (8) statistical results related to the gut microbiome. These were reported in Table 1.

**Table 1:** Summary of data used for analyses in the 26 articles

Study	Country	No. of controls (GI status)	Control: mean age ± SD; age range	No. of cases analysed (GI status)	Case: Mean age ± SD; age range	ASD and behavioural tools	Bacterial genome source	Methods
Adams et al. [30]	USA	39 HC (− GI)	7.7 ± 4.4; 2.5–18	58 (± GI)	6.9 ± 3.4; 2.5–18	ATEC	Fecal	Cultures, Vitek^®^ 2 microbial identification system
De Angelis et al. [31]	Italy	10 SIB (− GI)	4–10	20 (− GI)	4–10	DSM-IV, ADI-R, CARS, ADOS	Fecal	Cultures, bTEFAP of the 16S rDNA and 16S rRNA
Finegold et al. [32]	USA	8 HC (n/a)	n/a	13 (+ GI)	n/a	n/a	Fecal Gastric and duodenal fluids	Cultures, 16S rRNA
Finegold et al. [33]	USA	7 SIB (± GI) 8 HC (± GI)	2–13	33 (± GI)	2–13	Expert evaluation	Fecal	bTEFAP of 16S rRNA gene at the DNA level
Finegold et al. [34]	USA	13 HC (− GI)	2–9	33 (± GI)	2–9	n/a	Fecal	Cultures, PCR for the main *C. perfringens* toxins, verified with DNA sequencing
Gondalia et al. [35]	Australia	None	n/a	28 (± GI)	4.8 ± 3.3; 2–14	CARS, expert evaluation	Fecal	Cultures
Gondalia et al. [36]	Australia	53 SIB (8% + GI)	2–12	51 (55% + GI)	2–12	CARS, expert evaluation	Fecal	bTEFAP Gray28F 5′TTTGATCNTGGCTCAG and Gray519r 5′ GTNTTACNGCGGCKGCTG, qPCR
Inoue et al. [37]	Japan	6 HC (− GI)	3–5	6 (− GI)	3–5	DSM-5, PARS, M-CHAT	Fecal Blood	16S rRNA, DNA microarray analysis of PBMC, qPCR
Iovene et al. [38]	Italy	33 HC (− GI)	7.3 ± 3.1	47 (70% + GI)	6.0 ± 2.8	DSM-IV-TR, ADI-R, CARS, ADOS	Fecal	Cultures, Vitek^®^ 2 microbial identification system
Kang et al. [39]	USA	20 HC (− GI)	8.3 ± 4.4; 3–16	20 (+ GI)	6.7 ± 2.7; 3–16	ADI-R, ADOS, ATEC, PDD-BI	Fecal	16S rRNA, bTEFAP 104F (59-GGCGVACGGGTGAGTA A-39) and 530R (59-CCGCNGCNGCT GGCAC-39)
Kang et al. [40]	USA	20 HC (− GI)	Age-matched	18 (+ GI)	7–16	ADI-R, CARS, SRS, ABC, PGI-III, VABS	Fecal	16S rRNA
Kang et al. [41]	USA	21 HC (± GI)	8.4 ± 3.4, 4–17	23 (± GI)	10.1 ± 4.1; 4–17	ADI-R, ADOS, ATEC, PDD-BI	Fecal	16S rRNA, 104F (59-GGCGVACGGGTGAGTAA-39) and 530R (59-CCGCNGCNGCTGGCAC-39),
Kushak et al. [42]	USA	19 HC (+ GI)	16.1 ± 1.3	21 (+ GI)	14.4 ± 1.1	DSM-IV	Duodenal biopsy	16S rRNA, Gray28F 50TTTGATCNTGGCTCAG and Gray519r 50 GTNTTACNGCGGCKGCTG
Liu et al. [43]	China	None	n/a	20 (n/a)	1–8	DSM-5, CARS, ABC, SRS	Fecal	16S rRNA, CD 38 and RORA mRNA, 338F 5′-ACTCCTACGGGAGGCAGCA-3′ and 806R 5′-GGACTACHVGGGTWTCTAAT-3′
Luna et al. [44]	USA	21 HC (71% + GI)	3–18, + GI 3–14, -GI	14 (+ GI)	4–13	SRS, ADOS	Blood biopsy from colon	V1V3 and V4 regions of the 16S ribosomal RNA
Parracho et al. [45]	UK	12 SIB (25% + GI) 10 HC (− GI)	6 ± 2.15, 2–10 6 ± 2.88, 3–12	58 (91% + GI)	7 ± 3.76; 3–16	n/a	Fecal	FISH
Parracho et al. [46]	UK	None	n/a	17 (± GI)	4–16	DBC-P	Fecal	FISH
Shaaban et al. [47]	Egypt	30 HC (− GI)	Age and gender-matched	30 (− GI)	7.1 ± 1.4; 5–9	DSM-5, ADOS, ADI-R, ATEC, expert evaluation	Fecal	qPCR
Son et al. [48]	USA	44 SIB (30% + GI)	10.0 ± 1.8 8–13	59 (42% + GI)	10.3 ± 1.8; 8–13	ADI-R, ADOS, CBCL	Fecal	qPCR, V1V2 and V1V3 regions of 16S rRNA
Song et al. [49]	USA	8 HC (n/a)	n/a	15 (n/a)	n/a	n/a	Fecal	TaqMan qPCR of 16srRNA
Strati et al. [50]	Italy	40 HC (27.5% + GI)	9.2 ± 7.9	40 (12.5% + GI)	11.1 ± 6.8	DSM-5, ADOS, ABC, CARS	Fecal	16S rRNA and ITS sequencing
Tomova et al. [51]	Slovakia	9 SIB (78% + GI) 10 HC (60% + GI)	5–17 2–11	10 (90% + GI)	2–9	ADI-R, CARS, expert evaluation	Fecal	qPCR
Wang et al. [52]	Australia	22 SIB (27% + GI) 9 HC (11% + GI)	12 ± 1 (4.6–18.4) 9.5 ± 1.3 (3.5–15.2)	23 ( 39% + GI)	10.3 ± 0.8; 3.1–17.3	CARS, DSM-IV	Fecal	qPCR
Wang et al. [53]	Australia	22 SIB (27% + GI) 9 HC (11% + GI)	3–19	23 ( 39% + GI)	3–19	CARS, DSM-IV	Fecal	qPCR
Williams et al. [54]	USA	7 HC (+ GI)	4.0 ± 1.1; 3.9–5.5	15 (+ GI)	4.5 ± 1.3; 3.5–5.9	DSM-IV-TR, ADI-R, shortened CPEA regression interview	Biopsy from ileum and cecum	16sRNA
Williams et al. [55]	USA	9 HC (+ GI)	n/a	23 (+ GI)	n/a	DSM-IV-TR symptoms, ADI-R, shortened CPEA regression interview	Biopsy from ileum and cecum	*Sutterella*-specific 16S rRNA, qPCR

ABC = Autism Behaviour Checklist; ADI-R = Autism Diagnostic Interview-Revised; ADOS = Autism Diagnostic Observation Schedule; ATEC = Autism Treatment Evaluation Checklist; b-TEFAP = Bacterial tag-encoded FLX amplicon pyrosequencing; CARS = Childhood Autism Rating Scale score; CBCL = Child Behavior CheckList; CPEA = Collaborative Programs of Excellence in Autism; DBC-P = Developmental Behavior Checklist—Parent/Primary Carer; DSM-IV = Diagnostic and Statistical Manual of Mental Disorders, 4th edition; DSM-IV-R = Diagnostic and Statistical Manual of Mental Disorders, 4th edition text revised; DSM-5 = Diagnostic and Statistical Manual of Mental Disorders, 5th edition; FISH = fluorescence in situ hybridization; GI = gastrointestinal symptoms; GI (+) = GI symptoms present in the group; GI (−) = GI symptoms not present in the group; HC = Health controls; M-CHAT = Modified Checklist for Autism in Toddlers; n/a = not available; PBMC = Peripheral blood mononuclear cells; PARS = Pediatric Anxiety Rating Scale; PDD-BI = Pervasive Developmental Disorder Behavioral Inventory; PDD-NOS = Pervasive Developmental Disorder, not otherwise specified = Mild; PGI = Parent Global Impression; Severe = Autistic Disorder; SIB = Siblings without ASD, SRS = Social Responsiveness Scale; qPCR = quantitative Polymerase chain reaction, RORA = RAR Related Orphan Receptor A; rRNA = ribonucleic acid; VABS = Vineland Adaptative Behavior Scales

### Quantification and statistical analysis

All reported outcomes were organized into tables showing detailed differences in the gut microbiome from the phylum down to the species between case subjects with ASD and control groups. When studies implemented interventions, we also noted differences reported before and after the interventions. Results of each study were summarized as increased, decreased, or no change in relative abundance (percentage), absolute abundance (counts), or variety of each microorganism. To ensure a rigorous review, observational data without inferential statistics were not included. For results with statistical comparisons, both positive and negative findings were reported regardless of significance. When inferential statistics were completed yet no *p-*values or 95% confidence intervals could be found, the authors of this review used the data generated by the original authors and conducted tests of statistical significance. The majority of these tests included chi-square tests of independence for non-parametric, categorical data, two-sample and/or paired *t*-tests, and one-way analysis of variance (ANOVA). These analyses were completed using IBM SPSS Statistics, Version 22 (SPSS Inc., Chicago, IL).

## Results

In total, 26 papers [30–55] were selected for this systematic review (PRISMA Fig. 1; search criteria Additional file 1: Table S1). Two studies [52, 53] belonged to the same cohort but were both included in the synthesis of this systematic review because the gut microbes examined were different.

A summary of the papers used is provided in Table 1, including methodological techniques and the rigor of how ASD had been diagnosed. The detailed results from each study are presented in different tables (Tables 2, 3, 4 and 5), grouped according to bacterial taxonomic classification for easy comparison.

**Table 2:**
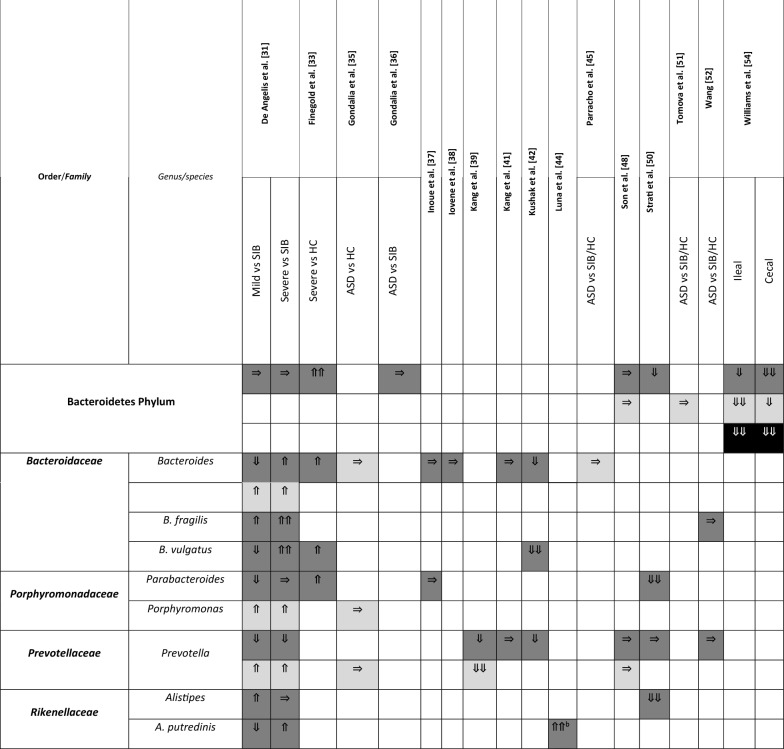
Changes in Bacteroidetes phylum between children with ASD and healthy controls

Arrows indicate whether the strains are increased (⇑), reported as no significant difference (⇒), or decreased (⇓) in (1) count (light grey), (2) percentage of the total microbiota (dark grey), and (3) variety (black); single arrow denoting *p* < 0.05, double arrows denoting *p* < 0.01

HC = healthy controls; SIB = sibling without ASD; Autistic Disorder = Severe; Pervasive Developmental Disorder, not otherwise specified (PDD-NOS) = Mild

^a^Described in text without definitive *p-*values

^b^Ages 13–18 only

^c^Ages below 6 only

**Table 3:**
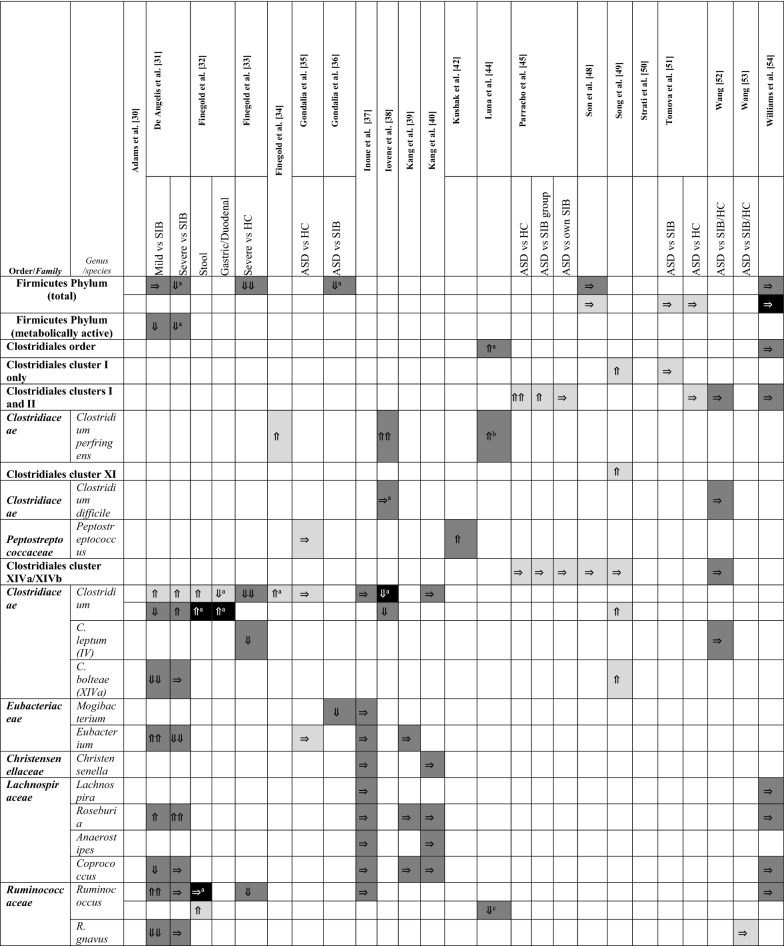

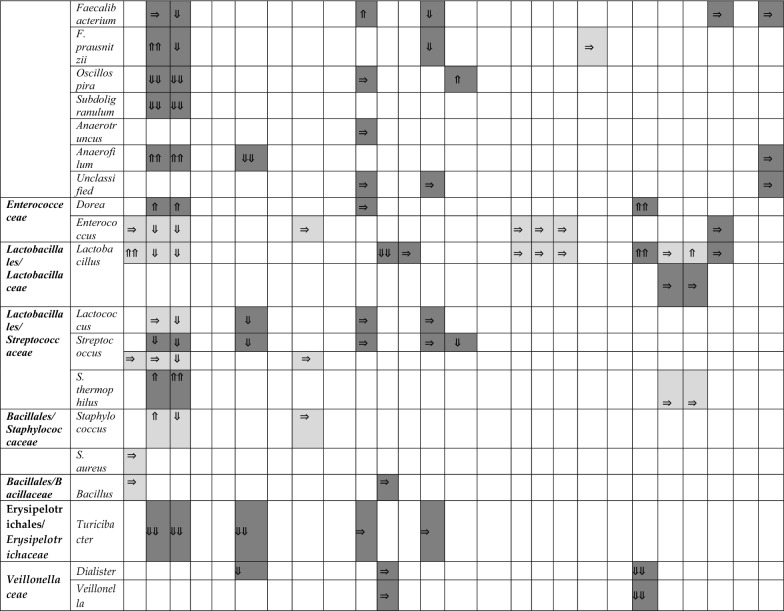
Changes in Firmicutes phylum between children with ASD and healthy controls

Arrows indicate whether the strains are increased (⇑), reported as no significant difference (⇒), or decreased (⇓) in (1) count (light grey), (2) percentage of the total microbiota (dark grey), and (3) variety (black); single arrow denoting *p* < 0.05, double arrows denoting *p* < 0.01

HC = healthy controls; SIB = sibling without ASD; Autistic Disorder = Severe; Pervasive Developmental Disorder, not otherwise specified (PDD-NOS) = Mild

^a^Described in text without definitive *p-*values

^b^Ages 13–18 only

^c^Ages below 6 only

**Table 4:**
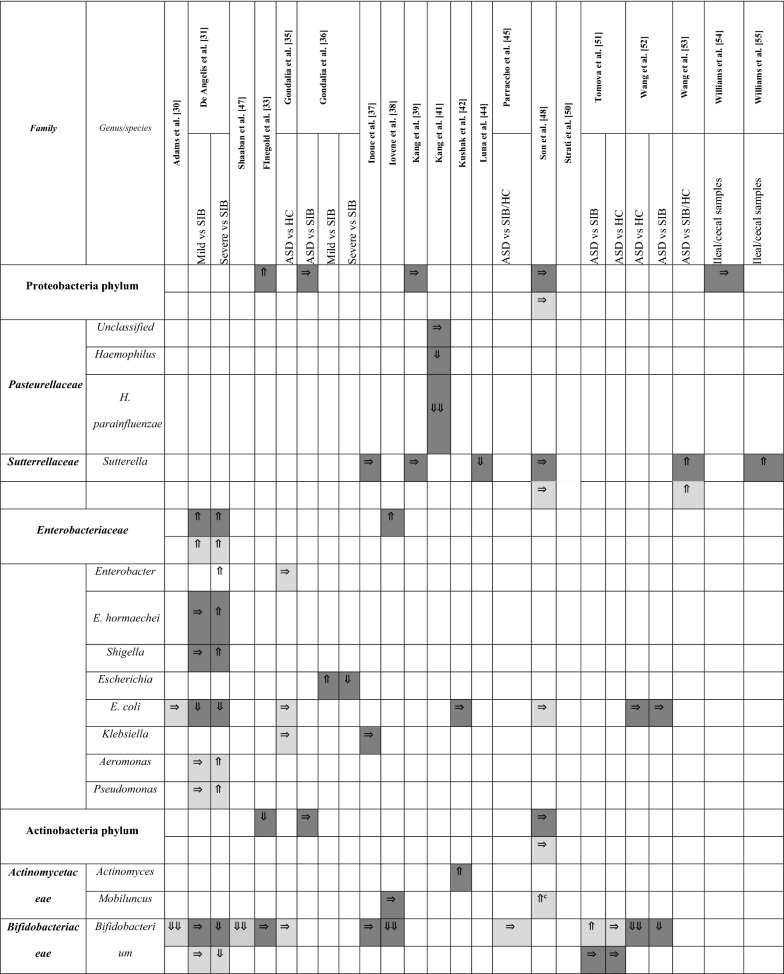

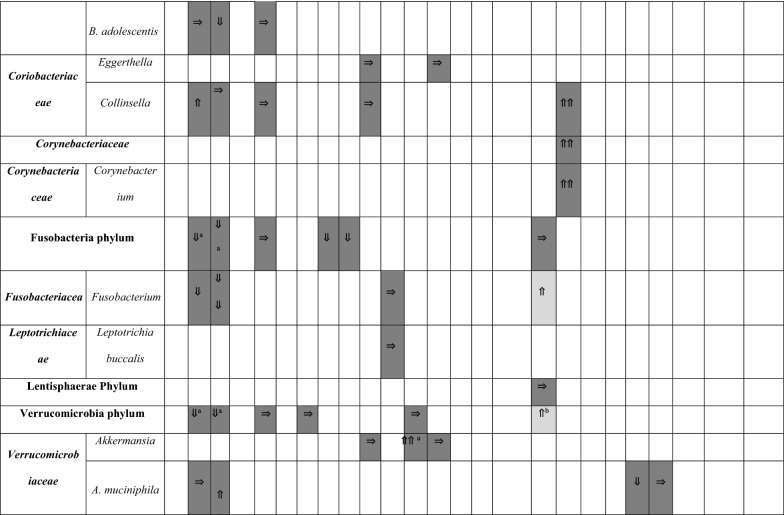
Changes in Proteobacteria, Actinobacteria, Fusobacteria, and Verrucomicrobia phyla between children with ASD and healthy controls.

Arrows indicate whether the strains are increased (⇑), reported as no significant difference (⇒), or decreased (⇓) in (1) count (light grey), (2) percentage of the total microbiota (dark grey), and (3) variety (black); single arrow denoting *p* < 0.05, double arrows denoting *p* < 0.01

HC = healthy controls; SIB = sibling without ASD; Autistic Disorder = Severe; Pervasive Developmental Disorder, not otherwise specified (PDD-NOS) = Mild

^a^Described in text without definitive *p-*values

^b^V1V2 datasets are statistically significant, but not V1V3

^c^V1V3 datasets are statistically significant, but not V1V2

**Table 5:**
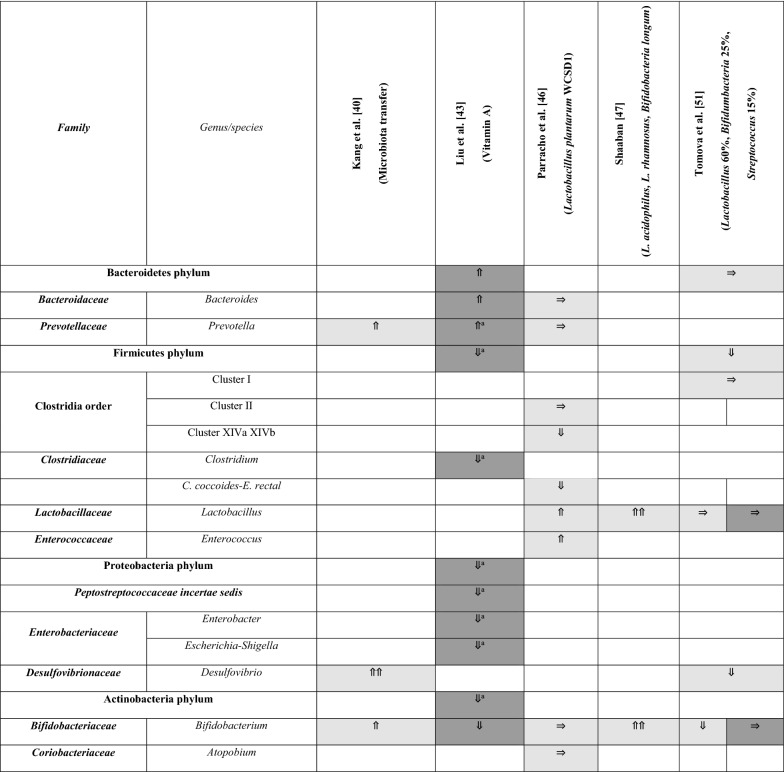
Changes in Bacteroidetes, Firmicutes, Proteobacteria, and Actinobacteria after intervention

Arrows indicate whether the strains are increased (⇑), reported as no significant difference (⇒), or decreased (⇓) in (1) count (light grey), (2) percentage of the total microbiota (dark grey), and (3) variety (black); single arrow denoting p < 0.05, double arrows denoting p < 0.01

HC = healthy controls; SIB = sibling without ASD; Autistic Disorder = Severe; Pervasive Developmental Disorder, not otherwise specified (PDD-NOS) = Mild

^a^Described in text without definitive p-values

### Changes to alpha and beta diversity

In metagenomics, alpha diversity represents the richness and the diversity of the microbiome in a single ecosystem. QIIME Operating taxonomic unit (OTU) counts, Chaos1, and Shannon index are commonly used to calculate alpha diversity. Based on the results of 11 papers that examined alpha diversity, there were no consistent patterns when comparing alpha diversity in children with ASD versus alpha diversity in siblings (SIB) and healthy controls (HC; Additional file 1: Table S2). Two studies showed increases [31, 33] and six studies showed no significant changes in alpha diversity [36, 42, 43, 48, 50, 54]. The last three studies indicated decreases; however, the studies came from one laboratory [39–41].

Beta diversity refers to the total variance in microbial community composition across varying environments. Bray–Curtis dissimilarity, Jaccard distance, and UniFrac are examples of indices used in the calculation. Among the seven [31, 36, 40–42, 48, 50] papers that reported on beta diversity, four [31, 40, 41, 50] papers showed significant differences in beta diversity between ASD and SIB/HC while the rest did not (Additional file 1: Table S2).

### Changes to Bacteroidetes

Six studies provided results on Bacteroidetes at the phylum level (Table 2). Only one study [33] demonstrated an increase in the percentage of Bacteroidetes in children with ASD, classified as “severe”, compared to HC (*p* = 0.001). Authors of this current review used data from this one study [33] and conducted *t*-tests to compare children labeled as “mild” against HC. We found that despite having “mild” ASD in the study, the percentage of Bacteroidetes in these children was still significantly increased compared to controls (*p* = 0.0012). The rest of the studies observed either a decrease in Bacteroidetes percentage [50, 54] or no significant differences between cases and controls [31, 36, 48].

Fifteen studies reported findings at the genus and/or species level of Bacteroidetes. Similar to the phylum Bacteroidetes as a whole, results from these studies were not consistent. The only genus with a more recognisable pattern was *Prevotella.* Seven out of fifteen studies described *Prevotella* and none of them showed a higher relative abundance in the stools of children with ASD when compared to controls. Instead, these seven studies showed either a non-significant result in relative abundance [41, 48, 50, 52] or a lower relative abundance in children classified as ASD versus SIB or HC [31, 39, 42]. There was no specific pattern in the absolute abundance of *Prevotella.* For all other genera and species (Table 2), studies generally contradicted each other and different laboratories found varying composition of Bacteroidetes species.

The other 24 detected species in the Bacteroidetes phyla were described in only one study each; hence, no summary could be made. However, we have included the results of all these species in Additional file 1: Table S3.

### Changes to Firmicutes

Table 3 shows changes in the phylum of Firmicutes. At the phylum level, none of the six studies showed an increase in absolute or relative abundance [31, 33, 36, 48, 51, 54]. The two studies on absolute abundance did not reveal any differences between ASD, SIB, and HC [48, 51]. For relative abundance in both total and metabolically active Firmicutes, the studies demonstrated either a decrease [31, 33, 36] or no significant differences between children with ASD, SIB, and HC [48, 51, 54].

Interestingly, from a Clostridiales cluster standpoint, the studies on Cluster I and Cluster II, as well as Cluster XI, while few, collectively suggested an increase in children with ASD [45, 49, 51, 52, 54]. Studies with negative findings were present, although none of the studies showed a decrease in these Clostridiales clusters. *Clostridium perfringens* at a species level had evidence for increased absolute and relative abundance in children with ASD versus typically developing children [34, 38, 44]. Cluster XIVa and XIVb, however, were quantitatively similar in percentage [52] and counts [45, 48, 49] in the available four studies. Many studies have focused on *Clostridium* as a genus and the method of addressing *Clostridium* quantity were not the same, with four studies using the number of counts, three using percentage of the total microbiota, and one using diversity within the genus as outcomes. The differences in methodology render the meta-analysis of these studies inconclusive.

In the families of *Eubacteriaceae*, *Christensenellaceae*, *Lachnospiraceae*, and *Ruminococcaceae*, the results were inconsistent. In the order Lactobacillales and family *Streptococcaceae*, *Streptococcus* results (not *S. thermophilus*) corroborated an overall decrease in counts and percentage in ASD cases when statistical significance was reached [30, 31, 33, 37, 41]. *Staphylococcus* species were not extensively studied.

An additional 83 species were reported in Additional file 1: Table S4. As there was a paucity of studies published on these species, no firm conclusions could be made.

### Changes in Proteobacteria, Enterobacteria, Actinobacteria, and other phyla

Members of the phylum Proteobacteria have a low abundance in the gut of healthy humans. However, several studies have observed correlations between an increase in abundance of Proteobacteria and diseased states [56]. As such, results from studies which measured changes to Proteobacteria were synthesised and presented in Table 4.

At the phylum level, no significant differences were observed in Proteobacteria between the two groups except one study indicated an increase in percentage [33]. Despite speculation that *Sutterella* was increased in children with ASD [53, 55], this was not true in every study. There were three studies suggesting no significant differences [38, 41, 48] and one suggesting a decrease [44], but this particular study included adolescents in their cohort.

There were insufficient studies examining the family *Enterobacteriaceae*. The general trend, however, supports no alterations in *E. coli* for children in ASD in five studies [30, 35, 42, 48, 52] vs one study [31] documenting a decrease in *E. coli* percentage among other microbiota. Actinobacteria as a phylum did not reveal any compelling results [33, 36, 48]; however, upon a closer look at *Bifidobacterium*, consistently lower counts and proportions were found in children with ASD versus their siblings [31, 52] or controls [30, 38, 47]. Only one study with a small sample size of 10 children with ASD contradicted this finding [51]. In this study, participants’ microbiome composition were likely different, as they were all from the eastern region of Central Europe and their diets might consist of different dairy products like sheep cheese and sour milk.

Fusobacteria phylum suggested a lower relative abundance in children with ASD vs unaffected siblings, although one study [48] demonstrated the opposite in terms of *Fusobacterium* absolute numbers. No specific findings were identified in the Verrucomicrobia phylum [31, 33, 36, 39, 48] and in the *Verrucomicrobiaceae* family [31, 37, 39, 41, 52].

Within these phyla, 42 additional bacterial species were described by the 26 studies, but were either unclassified or only measured by one study. We have listed positive and negative findings of each species in Additional file 1: Table S5. However, no comparisons or conclusions could be made from the limited number of studies on each species.

### Intervention studies involving a change in abundance of bacterial subtypes

The growing focus on the gut-brain axis led many researchers to perform studies which aimed to identify ways where the reversal of alterations in the gut microbiome could produce therapeutic effects on ASD symptoms, such as by administering probiotics or by changing diets of children with ASDs.

One study investigated the effect of administering vitamin A [43] on children with ASD and found an increase in abundance on the Bacteroidetes phylum level (Table 5). Both *Bacteroides* species and *Prevotella* species levels increased post-treatment. On the other hand, all of the other phyla and genera measured showed a decrease in abundance. Post-vitamin A administration, significant increases were also seen in other forms of biomarkers, including plasma retinol, CD38 and RORA mRNA levels. However, no changes were seen in the ASD symptomatology of the study participants [43].

Three other studies [46, 47, 51] examined the effects of probiotics on gut microbiome composition. The first one [46] showed that probiotics consisting of *Lactobacillus plantarum* WCSD1 decreased the bacterial counts of Clostridium clusters XIVa and XIVb and increased *Lactobacillus* species. However, behavioral improvements on ASD children, as reported on the Development Behaviour Checklist, were not significantly different between the probiotic feeding and placebo regimes. In another study where probiotics consisting of *L. acidophilus*, *L. rhamnosus*, and *Bifidobacteria longum* were used, both *Lactobacillus* and *Bifidobacterium* species increased [47]. Contrary to the first study, significant improvements in the severity of autism, as assessed by the Autism Treatment Evaluation Checklist (ATEC), were found in this second study. In the third study involving probiotic supplementation with Lactobacillus 60%, Bifidumbacteria 25%, Streptococcus 15% [51], a decrease in Firmicutes phylum and *Desulfovibrio* was observed after probiotics intervention. Surprisingly, a decrease in *Bifidobacterium* was found after probiotics and no significant changes were found in the *Lactobacillus* species, which contradicted aforementioned studies [43, 47, 52]. This third study did not describe the therapeutic effects of probiotics on ASD behaviors [51].

Microbiota transfer therapy (MTT) was also performed in a recent study and bacterial composition before and after MTT treatment were collected. After MTT intervention, an increase in the absolute abundance of *Prevotella*, *Bifidobacterium,* and *Desulfovibrio* species was observed. Additionally, clinical assessments showed that behavioral ASD symptoms improved significantly and remained improved 8 weeks after treatment ended [40].

Comparing changes in species across the various interventional studies, only *Lactobacillus* and *Prevotella* species showed a more consistent increase after probiotics interventions. However, it is important to note that each study used probiotics of different bacterial species. As such, purposeful conclusions cannot be drawn directly for comparison.

## Discussion

In the works reviewed, children diagnosed with ASD have various forms of dysregulation of the microbiome when compared to siblings or unrelated children without the ASD profile. Since each individual study describes a restricted and different bacterial composition, direct comparison between strains with similar classification is limited. However, the data follow a more consistent pattern for a few strains. Relative and absolute Clostridia clusters I, II, and XI are not found to be decreased in the gut microbiome of children with ASD when compared to those without. Similarly, the relative and absolute abundances of Firmicutes at the phylum level, *Streptococcus* at the genus level, *Prevotella* species, and *Bifidobacterium* species are not increased in children with ASD versus non-sibling controls. Of note, in all studies reviewed including intervention ones, the absolute abundance of *Bifidobacterium* species is significantly decreased in children with ASD compared to non-sibling controls, and the species is also significantly increased after intervention. Despite some recognizable patterns, the majority of microorganisms reviewed from phyla to species have disparate results across different studies. Hence, to date, gut microbial composition by itself does not provide a predictive biomarker for ASD and the single technology of high-throughput sequencing will need to be integrated with multiple sources of omics data (e.g. proteomics, transcriptomics, metabolomics, microRNAs and exosomes) to produce potential signatures for the spectrum of symptoms in individuals with ASD.

Although a direct causal mechanism of microbiome in the etiology of ASD in humans cannot be validated at this time, the gut microbiome likely alters brain functions through various other mechanisms, including environmental factors (e.g. in utero exposure to infection, maternal conditions, and medications), host genetics, host immune response regulation [12, 57, 58], excretion of metabolites such as tyrosine analogues, p-cresol, 4-ethylphenylsulfate, indoles, lipopolysaccharides and free amino acids [59-62], regulation of neurotransmitters and their receptors [21, 63], or neuroactive compounds [61, 62, 64].

Alterations of the host immune responses by gut microbiota are closely linked to ASD-related symptoms. The implicated cytokine pathways include, and not limited to, IL-5, IL-15, IL-17, IL-17a, IL-10, IL-1b, TNF-α, TGF-β1 and IFNγ [12, 18, 65, 66]. Interestingly, the gut microbiota has recently been shown to influence the immune system directly via activation of the vagus nerve [67, 68]. Furthermore, gut microbiota-derived short-chain fatty acids (SCFAs), such as propionic acid [69, 70] and butyric acid [71, 72], produced by bacterial fermentation of carbohydrates have immunomodulatory properties, e.g. upregulating genes associated with immune activation [69], regulating T cells and cytokine production [70], microglia homeostasis during developmentally sensitive periods [73], and neuronal excitability [74], and have recently been used in vivo in the treatment of inflammatory conditions such as inflammatory bowel diseases [75]. In addition to understanding microbiome composition differences in children with ASD, there is a need to investigate the patterns of dysregulation in their immune responses as well as to look more upstream at the maternal immune response during pregnancy. Prior literature has substantiated that infections during pregnancy have been correlated with increased frequency of neurodevelopmental disorders in offspring [16, 17, 76–78]. Specifically, there is an association between ASD and maternal infection requiring hospitalization during pregnancy, elevated C-reactive protein, and a family history of autoimmune diseases. Thus, future studies will need to explain the bidirectional and possibly transgenerational roles of microbiome alterations and immune pathways on behaviours.

A promising development in this field points to the need to consider interactions between host genetics and microbial composition. Differences in microbiome diversity have been shown to be partially attributed by genotype and sex [79–83]. In a rodent model, Tabouy et al. [84] used the Shank3 KO mice and demonstrated that specific bacterial species (i.e. *L. reuteri*) were sensitive to an autism-related mutation, were decreased in abundance, and positively correlated with the expression of gamma-aminobutyric acid (GABA) receptor in the brain. Treatment with *L. reuteri* resulted in an increase of both GABA receptor gene expression and protein levels in brain regions of mice, which also corresponded to improvements in social engagement. It is noteworthy to mention that there is a paucity of research examining the interactions of host genetics and microbial dysregulation in humans with ASD. Perhaps it is worthwhile to isolate individuals with the same autism-related genotype and investigate for potential dysbiosis in their microbiome, along with changes in gene expression and/or in brain structure. Likewise, studies suggesting therapeutic potential for probiotic treatment has currently looked at individuals with the ASD profile as a whole. Future studies may consider subgroup analysis (e.g. responders vs non-responders) to understand the potential differences between subgroups.

Lastly, the gut microbiome’s contribution to neurological development and regulation has been implicated and demonstrated in animal models [85]. For example, gnotobiotic animals demonstrate heightened hypothalamic-pituitary response, elevated plasma adrenocorticotropic hormone and corticosterone, and reduced brain-derived neurotrophic factor (BDNF) expression levels in the cortex and hippocampus [86]. Absence of colonization results in differential expression of proteins involved in synaptogenesis [87] and atypical development [88]. Subsequent microbial colonization reverses these processes. Furthermore, gut microbiota manufactures neuroactive chemicals and influences levels of circulating 5-hydroxytryptamine (5-HT) and serotonin, thereby altering fetal neuronal cell synaptogenesis [89] and neuronal morphogenesis [90], respectively. Although mounting evidence is accumulating for microbiome’s role in neural development, the precise nature of how multiple systems interact or overlap remain poorly defined.

The variety of protocols for sampling and characterization of microbial ecology among included studies also warrant discussion. Since the human microbiome exhibits considerable spatial and temporal variability, single samples obtained from a specific anatomical site may not be representative of its true diversity at any given time and may especially fail to capture rarer or less abundant taxa. Heterogeneity also exists with regards to workflows for specimen storage and processing, and factors such as shipping time and ambient temperature are established to influence the microbial composition in poorly-handled specimens. In terms of experimental procedures, high-throughput nucleic acid-based interrogation represent the most common technique used in included studies. However, interpretation of the collective results across studies may be constrained by the lack of standardization of experimental protocols and is further hampered by suboptimal inter-platform agreement and measurement reliability. Finally, with regards to the comparison of microbial constituents between ASD cases and controls, the issue of multiple testing looms large. For these and other reasons, it is essential that the salient findings summarized in the present review are externally validated by independent laboratories.

Autism spectrum disorder is a neurobiological disorder which is potentially a result of disruptions in normal brain growth very early in development. The studies reviewed have not reported on the birth or pre-diagnosis microbiome of children with ASD. Instead, studies generally report bacterial diversity after children are diagnosed with ASD. It is hard to determine the directionality of the association between microbiome differences and dietary habits. It is possible that children with ASD have greater likelihood of having more unique preferences in certain diets and this limited diet variety may account for microbiome differences. One study suggests that children with ASD may have an increased intake of chia seeds in smoothies, which is associated with specific microbiome findings [48]. Children with ASD are also sometimes placed on non-specific gluten-free, casein-free diets, which easily change one’s gut microbiome composition.

The literature currently lacks prospective studies that follow a child from prior to ASD diagnosis, preferably as an infant, with repeated objective assessment of ASD symptomatology and its trajectory at the same time as stool collection for microbiome. Given the long duration of such prospective studies, it is unlikely that the same environmental conditions such as diet, exposure to antibiotics or other medications, pets in the home, exposure to livestock, and limits on travel may be imposed on the participants, which will further complicate interpretation of microbial samples. Nonetheless, ongoing investigations, such as the National Institutes of Health (NIH) Environmental influences on Child Health Outcomes (ECHO) study, have already started the collection of infant microbiotas with planned serial samples. When these studies are complemented with mechanistic experiments in animal models, they can be powerful in giving insight into human biology.

Research studies of this kind requires the involvement of professionals with clinical expertise in children with ASD. In this review, only a few studies have involved developmental specialists and psychologists who are apt in monitoring changes in ASD symptoms [32, 48]. Parent-reported questionnaires, while important to provide a summary of behaviors within the home setting, are not as objective compared to experienced observations in standardized assessments by psychologists or developmental-behavioral pediatricians. The heterogeneous nature of ASD is also a challenge in review studies. Further, the diagnostic criteria for ASD and classification of ASD into subtypes have been updated in 2013. Older studies classifying children into Asperger Disorder, PDD-NOS, and Autistic Disorder are based on the older edition of DSM-IV and not the DSM-5. There are studies to support that these diagnoses do not translate directly to an ASD diagnosis on DSM-5 [1, 91]. Future studies should consider a rigorous diagnosis of ASD and a description of the variety of ASD symptomatology in the participants, along with documentation of diet, intake of probiotics, antibiotics, travels, and episodes of gastrointestinal symptoms.

In summary, we provide data to show that the current literature on dysbiosis in children with ASD does not provide a predictive signature for the condition or symptoms. However, researchers may take note of the general consistencies found in composition changes of *Prevotella*, Firmicutes as a whole, three Clostridia clusters, *C. perfringens,* and *Bifidobacterium* in children with ASD to design future studies and to look deeper into the influence of these microorganisms on multi-system pathways.

The relationship of the microbiome and social behaviors is multifaceted and complex involving not only environmental factors and immune responses, but also the genetic background of the host. Further suggestions for future research include confirming the potential therapeutic qualities of specific microbial reconstitution in humans, dissecting the overlapping pathways between the microbiome and various organ systems, as well as the use of microbial metabolome and other omics platforms to study this topic.

## Supplementary information

**Additional file 1: Table S1.** Search criteria, inclusion and exclusion criteria. **Table S2.** Differences between alpha and beta diversities. **Table S3.** Bacteroidetes phylum, single observation studies. **Table S4.** Firmicutes phylum, single observation studies. **Table S5.** Proteobacteria phylum, single observation studies.

## Data Availability

The study dataset was generated using the 26 articles stated in Table 1. The datasets supporting the current study were extracted by authors of this review and they are all shown in the published tables.

## Abbreviations

AD: Autistic Disorder
ASD: autism spectrum disorder
BDNF: brain-derived neurotrophic factor
DSM-IV: Diagnostic and Statistical Manual for Mental Disorder—Fourth Edition
ECHO: Environmental influences on Child Health Outcomes
GABA: gamma-aminobutyric acid
HC: healthy controls
MIA: maternal immune activation
MTT: microbiota transfer therapy
NIH: National Institutes of Health
ANOVA: one-way analysis of variance
OTU: operating taxonomic unit
PDD-NOS: Pervasive Developmental Disorder, not otherwise specified
PRISMA: Preferred Reporting Items for Systematic Reviews and Meta-analyses
SIB: siblings
SCFAs: short-chain fatty acids
5-HT: 5-hydroxytryptamine
