# Supplementary material for: Gut microbiota changes in children with autism spectrum disorder: a systematic review
Source: Gut Pathog. 2020 Feb 3;12:6. doi: 10.1186/s13099-020-0346-1 (PMC6996179; doi:10.1186/s13099-020-0346-1)
Supplement: Supplementary file 1 — Additional file 1: Table S1. Search criteria, inclusion and exclusion criteria. Table S2. Differences between alpha and beta diversities. Table S3. Bacteroidetes phylum, single observation studies. Table S4. Firmicutes phylum, single observation studies. Table S5. Proteobacteria phylum, single observation studies. [file 13099_2020_346_MOESM1_ESM.docx]

**Additional Table S1.** Search criteria, inclusion and exclusion criteria

| **Search criteria** | |
| --- | --- |
| **Topic** | **Search terms** |
| Gut microbiota and neurological behaviour | ("gastrointestinal microbiome"[MeSH Terms] OR ("gastrointestinal"[All Fields] AND "microbiome"[All Fields]) OR "gastrointestinal microbiome"[All Fields] OR ("gut"[All Fields] AND "microbiota"[All Fields]) OR "gut microbiota"[All Fields]) AND neurological[All Fields] AND ("behaviour"[All Fields] OR "behavior"[MeSH Terms] OR "behavior"[All Fields]) |
| Microbiota-gut-brain-axis | microbiota-gut-brain[All Fields] AND ("axis, cervical vertebra"[MeSH Terms] OR ("axis"[All Fields] AND "cervical"[All Fields] AND "vertebra"[All Fields]) OR "cervical vertebra axis"[All Fields] OR "axis"[All Fields]) |
| Gut microbiota and neuropsychiatric disorders | ("gastrointestinal microbiome"[MeSH Terms] OR ("gastrointestinal"[All Fields] AND "microbiome"[All Fields]) OR "gastrointestinal microbiome"[All Fields] OR ("gut"[All Fields] AND "microbiota"[All Fields]) OR "gut microbiota"[All Fields]) AND neuropsychiatric[All Fields] AND ("disease"[MeSH Terms] OR "disease"[All Fields] OR "disorders"[All Fields]) |
| Gut microbiota and effects on child brain | ("gastrointestinal microbiome"[MeSH Terms] OR ("gastrointestinal"[All Fields] AND "microbiome"[All Fields]) OR "gastrointestinal microbiome"[All Fields] OR ("gut"[All Fields] AND "microbiota"[All Fields]) OR "gut microbiota"[All Fields]) AND effects[All Fields] AND ("child"[MeSH Terms] OR "child"[All Fields]) AND ("brain"[MeSH Terms] OR "brain"[All Fields]) |
| Gut microbiota and children and behaviour | ("gastrointestinal microbiome"[MeSH Terms] OR ("gastrointestinal"[All Fields] AND "microbiome"[All Fields]) OR "gastrointestinal microbiome"[All Fields] OR ("gut"[All Fields] AND "microbiota"[All Fields]) OR "gut microbiota"[All Fields]) AND ("child"[MeSH Terms] OR "child"[All Fields] OR "children"[All Fields]) AND ("behaviour"[All Fields] OR "behavior"[MeSH Terms] OR "behavior"[All Fields]) |
| Gut microbiota and cognitive development | ("gastrointestinal microbiome"[MeSH Terms] OR ("gastrointestinal"[All Fields] AND "microbiome"[All Fields]) OR "gastrointestinal microbiome"[All Fields] OR ("gut"[All Fields] AND "microbiota"[All Fields]) OR "gut microbiota"[All Fields]) AND ("Cogn Dev"[Journal] OR ("cognitive"[All Fields] AND "development"[All Fields]) OR "cognitive development"[All Fields]) |
| Gut microbiota and brain development | ("gastrointestinal microbiome"[MeSH Terms] OR ("gastrointestinal"[All Fields] AND "microbiome"[All Fields]) OR "gastrointestinal microbiome"[All Fields] OR ("gut"[All Fields] AND "microbiota"[All Fields]) OR "gut microbiota"[All Fields]) AND ("brain"[All Fields] AND "development"[All Fields]) OR "brain development"[All Fields] OR "Brain Dev"[Journal] OR ("brain"[All Fields] AND "development"[All Fields]) OR "brain development"[All Fields]) |
| Gut microbiota and brain development and children behaviour | ("gastrointestinal microbiome"[MeSH Terms] OR ("gastrointestinal"[All Fields] AND "microbiome"[All Fields]) OR "gastrointestinal microbiome"[All Fields] OR ("gut"[All Fields] AND "microbiota"[All Fields]) OR "gut microbiota"[All Fields]) AND ("No To Hattatsu"[Journal] OR ("brain"[All Fields] AND "development"[All Fields]) OR "brain development"[All Fields] OR "Brain Dev"[Journal] OR ("brain"[All Fields] AND "development"[All Fields]) OR "brain development"[All Fields]) AND ("child"[MeSH Terms] OR "child"[All Fields] OR "children"[All Fields]) AND ("behaviour"[All Fields] OR "behavior"[MeSH Terms] OR "behavior"[All Fields]) |
| Gut microbiota and brain function | ("gastrointestinal microbiome"[MeSH Terms] OR ("gastrointestinal"[All Fields] AND "microbiome"[All Fields]) OR "gastrointestinal microbiome"[All Fields] OR ("gut"[All Fields] AND "microbiota"[All Fields]) OR "gut microbiota"[All Fields]) AND ("brain"[MeSH Terms] OR "brain"[All Fields]) AND ("physiology"[Subheading] OR "physiology"[All Fields] OR "function"[All Fields] OR "physiology"[MeSH Terms] OR "function"[All Fields]) |

**Additional Table S2.** Changes in alpha and beta diversity and the indices used

| **Journal article** | **Alpha diversity** | **Beta diversity** | **Indices used** | | | | | | | | | | | | | |
| --- | --- | --- | --- | --- | --- | --- | --- | --- | --- | --- | --- | --- | --- | --- | --- | --- |
|  |  |  | **ACE** | **Bray-Curtise** | **Chao1** | **Faith** | **Good's coverage** | **Jaccard** | **Morisita-Horn** | **Peilou** | **Phylogenetic diversity** | **Simpson** | **Shannon** | **Rarefraction** | **UniFrac PCoA** | **QIIME** |
| Adams J.B. et al., 2011 (26) | n/a | n/a |  |  |  |  |  |  |  |  |  |  |  |  |  |  |
| De Angelis M. et al., 2013 (27) | ⇑ ASD (Ref: SIB) | Different between ASD vs HC^a^ |  |  | X |  | X |  |  |  |  |  | X | X |  | X |
| El Gendy, 2016 (28) | n/a | n/a |  |  |  |  |  |  |  |  |  |  |  |  |  |  |
| Finegold S.M. et al., 2002 (29) | n/a | n/a |  |  |  |  |  |  |  |  |  |  |  |  |  |  |
| [Finegold S.M. et al., 2010](about:blank#_msocom_5) (30) | ⇑ ASD/SIB (Ref: HC) | n/a | X |  | X |  |  |  |  |  |  |  |  | X |  |  |
| Finegold S.M. et al., 2017 (31) | n/a | n/a |  |  |  |  |  |  |  |  |  |  |  |  |  |  |
| Gondalia S.V. et al., 2010 (32) | n/a | n/a |  |  |  |  |  |  |  |  |  |  |  |  |  |  |
| Gondalia S.V. et al., 2012 (33) | ⇒ ASD (Ref: HC) | ⇒ ASD (Ref: HC) |  |  | X |  |  |  |  |  |  |  |  | X | X |  |
| Inoue R. et al., 2016 (34) | n/a | n/a |  |  |  |  |  |  |  |  |  |  |  |  |  |  |
| Iovene M.R. et al., 2016 (35) | n/a | n/a |  |  |  |  |  |  |  |  |  |  |  |  |  |  |
| Kang D.-W. et al., 2013 (36) | ⇓ ASD (Re: HC) | n/a |  |  | X |  |  |  |  |  | X |  | X | X |  |  |
| Kang D.-W. et al., 2017 (37) | ⇓ ASD (Re: HC) | Different between ASD vs SIB^a^ |  |  |  | X |  |  |  |  | X |  |  | X | X |  |
| Kang D.-W. et al., 2018 (38) | ⇓ ASD (Re: SIB) | Different between ASD vs SIB^a^ |  | X |  | X |  |  |  | X | X |  | X | X | X | X |
| Kushak R.I. et al., 2017 (39) | ⇒ ASD (Ref: HC) | ⇒ ASD (Ref: HC) |  |  | X |  |  |  |  |  |  |  | X |  | X |  |
| [Liu J. et al., 2017](about:blank#_msocom_9) (40) | ⇒ ASD (Ref: n/a) | n/a | X |  | X |  |  |  |  |  |  | X | X |  |  |  |
| Luna R.A. et al., 2017 (41) | n/a | n/a |  |  |  |  |  |  |  |  |  |  |  |  |  |  |
| Parracho H. et al., 2005 (42) | n/a | n/a |  |  |  |  |  |  |  |  |  |  |  |  |  |  |
| Parracho H. et al., 2010 (43) | n/a | n/a |  |  |  |  |  |  |  |  |  |  |  |  |  |  |
| Son J.S. et al., 2015 (44) | ⇒ ASD (Ref: SIB) | ⇒ ASD (Ref: SIB) |  | X | X |  |  | X | X |  |  |  | X |  |  |  |
| Song Y. et al., 2004 (45) | n/a | n/a |  |  |  |  |  |  |  |  |  |  |  |  |  |  |
| Strati F. et al., 2017 (46) | ⇒ ASD (Ref: HC) | Different between ASD vs control^a^ |  | X |  |  |  |  |  |  |  |  |  |  | X |  |
| Tomova A. et al., 2015 (47) | n/a | n/a |  |  |  |  |  |  |  |  |  |  |  |  |  |  |
| Wang L. V. et al., 2011 (48) | n/a | n/a |  |  |  |  |  |  |  |  |  |  |  |  |  |  |
| Wang L.V. et al., 2013 (49) | n/a | n/a |  |  |  |  |  |  |  |  |  |  |  |  |  |  |
| Williams B.L. et al, 2011 (50) | ⇒ ASD (Ref: HC) | n/a |  |  |  |  |  |  |  |  |  |  | X | X |  |  |
| Williams B.L. et al., 2012(51) | n/a | n/a |  |  |  |  |  |  |  |  |  |  |  |  |  |  |
| **Footnote**: Arrows indicate whether the diversity is **increased** (⇑), reported as **no significant difference** (⇒), or **decreased** (⇓); single arrow denoting *p* < 0.05, double arrows denoting *p* < 0.01. ^a^Unknown direction | | | | | | | | | | | | | | | | |

**Additional Table S3.** Changes in Bacteroidetes phylum between children with ASD and healthy controls (Single studies)

| **Order**/***Family*** | *Genus/species* | **De Angelis et al 2013 (27)** | | **Finegold et al 2010 (30)** | **Gondalia et al 2010 (32)** | **Inoue et al 2016 (34)** | **Iovene et al 2016 (35)** | **Kang et al 2013 (36)** | **Kang et al 2018 (38)** | **Luna et al 2017 (41)** | **Strati et al 2017 (46)** |
| --- | --- | --- | --- | --- | --- | --- | --- | --- | --- | --- | --- |
|  |  | Mild vs SIB | Severe vs SIB | Severe vs HC | ASD vs HC |  |  |  |  |  |  |
| **Bacteroidales Order** | |  |  |  |  |  |  |  |  |  | ⇓⇓ |
| ***Bacteroidaceae*** | *Bacteroides coprocola* | ⇑ | ⇓ |  |  |  |  |  |  |  |  |
|  | *B. faecis* | ⇒ | ⇑ |  |  |  |  |  |  |  |  |
|  | *B. intestinalis* | ⇒ | ⇑ |  |  |  |  |  |  |  |  |
|  | *B. massiliensis* | ⇑ | ⇓ |  |  |  |  |  |  |  |  |
|  | *B. ovatus* | ⇓ | ⇒ |  |  |  |  |  |  |  |  |
|  | *B. uniformis* | ⇓ | ⇓ |  |  |  |  |  |  |  |  |
|  | *Unclassified* |  |  |  |  |  |  |  | ⇒ |  |  |
| ***Marinilabiliaceae*** | *Alkaliflexus* |  |  | ⇑⇑ |  |  |  |  |  |  |  |
| ***Odoribacteraceae*** | *Odoribacter* |  |  |  |  |  |  |  | ⇒ |  |  |
|  | *O. splanchnicus* | ⇒ | ⇒ |  |  |  |  |  |  |  |  |
|  | *Butyricimonas* |  |  |  |  |  |  | ⇒ |  |  |  |
| ***Porphyromonadaceae*** | *Barnesiella* |  |  |  |  |  |  |  | ⇒ |  |  |
|  | *B. intestinihominis* | ⇓ | ⇑ |  |  |  |  |  |  |  |  |
|  | *Eurobacterium* |  |  |  | ⇒ |  |  |  |  |  |  |
|  | *Parabacteroides distasonis* |  |  |  |  |  |  |  |  | ⇓^a^ |  |
|  | *Unclassified* |  |  |  |  | ⇒ |  |  |  |  |  |
| ***Prevotellaceae*** | |  |  |  |  |  |  | ⇒ |  |  |  |
| ***Prevotellaceae*** | *Prevotella copri* | ⇓⇓ | ⇑ |  |  |  |  |  |  |  |  |
|  | *P. intermedia* |  |  |  |  |  | ⇒ |  |  |  |  |
|  | *Paraprevotella* |  |  |  |  |  |  | ⇒ |  |  |  |
|  | *Unclassified* |  |  |  |  |  |  | ⇒ |  |  |  |
| ***Rikenellaceae*** | *Alistipes indistinctus* | ⇒ | ⇑ |  |  |  |  |  |  |  |  |
|  | *A. onderdonkii* | ⇑ | ⇓ |  |  |  |  |  |  |  |  |
|  | *A. shahii* | ⇑ | ⇑ |  |  |  |  |  |  |  |  |
|  | *Unclassified* |  |  |  |  | ⇒ |  | ⇒ | ⇒ |  |  |
| **Footnote**: Arrows indicate whether the strains are **increased** (⇑), reported as **no significant difference** (⇒), or **decreased** (⇓) in  1) **count** (light grey), 2) **percentage of the total flora** (dark grey), and 3) **variety** (black); single arrow denoting *p*<0.05, double arrows denoting *p* <0.01. ^a^Ages 13-18 only. HC = healthy controls; SIB = sibling without ASD; Autistic Disorder=Severe; Pervasive Developmental Disorder, not otherwise specified (PDD-NOS)=Mild | | | | | | | | | | | |

**Additional Table S4.** Changes in Firmicutes phylum between children with ASD and healthy controls (Single studies)

| ***Family*** | Genus/species | **De Angelis et al 2013 (27)** | | **Finegold et al 2010 (30)** | **Gondalia et al 2012 (33)** | | **Inoue et al 2016 (34)** | **Iovene et al 2017 (35)** | **Kang et al 2013 (36)** | **Kang et al 2018 (38)** | **Luna et al 2017 (41)** | **Son et al 2015 (44)** | **Song et al 2004 (45)** | **Strati et al 2017 (46)** | **Wang et al 2013 (49)** | **Williams et al 2011 (50)** |
| --- | --- | --- | --- | --- | --- | --- | --- | --- | --- | --- | --- | --- | --- | --- | --- | --- |
|  |  | Mild vs SIB | Severe vs SIB | Severe vs HC | Mild vs SIB | Severe vs SIB |  |  |  |  |  |  | ASD vs SIB |  | ASD vs HC/SIB |  |
| ***Clostridiaceae* (cluster XIVa species)** | *Clostridium aldenense* |  |  |  |  |  |  |  |  |  | ⇑ |  |  |  |  |  |
|  | *C. aminophilum* | ⇒ | ⇑ |  |  |  |  |  |  |  |  |  |  |  |  |  |
|  | *C. asparagiforme* | ⇒ | ⇑⇑ |  |  |  |  |  |  |  |  |  |  |  |  |  |
|  | *C. lituseburense* |  |  |  |  |  |  |  |  |  | ⇑⇑ |  |  |  |  |  |
|  | *C. nexile* | ⇓ | ⇓ |  |  |  |  |  |  |  |  |  |  |  |  |  |
| ***Lachnospiraceae*** | |  |  |  |  |  |  |  |  |  |  |  |  |  |  | ⇒ |
|  | *Roseburia faecis* | ⇓⇓ | ⇓ |  |  |  |  |  |  |  |  |  |  |  |  |  |
|  | *R. inulinivorans* | ⇑ | ⇑⇑ |  |  |  |  |  |  |  |  |  |  |  |  |  |
|  | *R. hominis* | ⇓ | ⇒ |  |  |  |  |  |  |  |  |  |  |  |  |  |
|  | *R. intestinalis* | ⇓ | ⇓ |  |  |  |  |  |  |  |  |  |  |  |  |  |
|  | *Coprococcus eutactus* | ⇓ | ⇒ |  |  |  |  |  |  |  |  |  |  |  |  |  |
|  | *Lachnospira pectinoschiza* | ⇓ | ⇒ |  |  |  |  |  |  |  |  |  |  |  |  |  |
|  | *“Lachnoclostridium hathewayi”(82)* |  |  |  |  |  |  |  |  |  | ⇑ |  |  |  |  |  |
|  | incertae sedis |  |  |  |  |  |  |  |  |  |  |  |  |  |  | ⇒ |
|  | *Unclassified* |  |  |  |  |  | ⇒ |  |  |  |  |  |  |  |  | ⇒ |
|  | *“Sporobacterium WAL 1855D” (83)* |  |  |  |  |  |  |  |  | ⇒ |  |  |  |  |  |  |
|  | *Lachnoclostridium bolteae* |  |  |  |  |  |  |  |  |  | ⇑ |  |  |  |  |  |
| ***Ruminococceae*** | |  |  |  |  |  |  |  |  |  |  |  |  |  |  |  |
|  | *Ruminococcus torques* |  |  |  |  |  |  |  |  |  |  |  |  |  | ⇒ |  |
|  | *R. obeum* | ⇒ | ⇓ |  |  |  |  |  |  |  |  |  |  |  |  |  |
|  | *Butyricoccus* |  |  |  |  |  |  |  |  | ⇒ |  |  |  |  |  |  |
|  | *Ethanoligenens* |  |  | ⇓ |  |  |  |  |  |  |  |  |  |  |  |  |
|  | *Sporobacter* | ⇒ | ⇒ |  |  |  |  |  |  |  |  |  |  |  |  |  |
|  | *S. termitidis* | ⇓ | ⇓ |  |  |  |  |  |  |  |  |  |  |  |  |  |
|  | *Clostridium aminophilum* | ⇒ | ⇑ |  |  |  |  |  |  |  |  |  |  |  |  |  |
| ***Eubacteriaceae*** | *Eubacterium coprostanoligenes* | ⇒ | ⇓ |  |  |  |  |  |  |  |  |  |  |  |  |  |
|  | *E. ventriosum* | ⇒ | ⇓ |  |  |  |  |  |  |  |  |  |  |  |  |  |
|  | *E. siraeum* | ⇒ | ⇑⇑ |  |  |  |  |  |  |  |  |  |  |  |  |  |
|  | *E. limosum* |  |  |  |  |  |  | ⇒ |  |  |  |  |  |  |  |  |
|  | *E. eligens* | ⇓ | ⇓ |  |  |  |  |  |  |  |  |  |  |  |  |  |
|  | *Anaerofustis* |  |  |  | ⇑ | ⇑ |  |  |  |  |  |  |  |  |  |  |
|  | *Pseudoramibacter* |  |  | ⇓ |  |  |  |  |  |  |  |  |  |  |  |  |
| ***Enterococcaceae*** | *Enterococcus casseliflavus* | ⇑ | ⇒ |  |  |  |  |  |  |  |  |  |  |  |  |  |
|  | *Blautia* |  |  |  |  |  | ⇓ |  |  |  |  |  |  |  |  |  |
|  | *Blautia luti* |  |  |  |  |  |  |  |  |  | ⇓ |  |  |  |  |  |
|  | *Dorea formicigenerans* |  |  |  |  |  |  |  |  |  | ⇓⇓ |  |  |  |  |  |
| **Clostridiales*/ Syntrophomonadaceae*** | *Syntrophomonas* |  |  |  | ⇓ | ⇑ |  |  |  |  |  |  |  |  |  |  |
| **Clostridiales/Peptostreptococceae** | *Helcococcus* |  |  | ⇓ |  |  |  |  |  |  |  |  |  |  |  |  |
|  | *Terrisporobacter* |  |  |  |  |  |  |  |  |  | ⇑ |  |  |  |  |  |
|  | *Unclassified* |  |  |  |  |  | ⇒ |  |  |  |  |  |  |  |  |  |
| ***Peptococcaceae*** | *Peptococcus* |  |  |  |  |  |  | ⇒ |  |  |  |  |  |  |  |  |
| **Clostridiales/ *Clostridiaceae*** | *Caloramator* | ⇒ | ⇑⇑ |  |  |  |  |  |  |  |  |  |  |  |  |  |
|  | *Acetanaerobacterium* |  |  | ⇑ |  |  |  |  |  |  |  |  |  |  |  |  |
|  | *Sarcina* | ⇒ | ⇑ |  |  |  |  |  |  |  |  |  |  |  |  |  |
|  | *S. ventriculi* | ⇒ | ⇑ |  |  |  |  |  |  |  |  |  |  |  |  |  |
|  | *Clostridium orbiscindens (Flavonifractor plautii)* | ⇒ | ⇑ |  |  |  |  |  |  |  |  |  |  |  |  |  |
|  | *C. asparagiforme* | ⇒ | ⇑⇑ |  |  |  |  |  |  |  |  |  |  |  |  |  |
|  | *C. symbiosum* | ⇒ | ⇑ |  |  |  |  |  |  |  |  |  |  |  |  |  |
|  | *C. methylpentosum* | ⇓ | ⇓ |  |  |  |  |  |  |  |  |  |  |  |  |  |
|  | *C. bartlettii* | ⇓ | ⇓ |  |  |  |  |  |  |  |  |  |  |  |  |  |
|  | *Bryantella (Marvinbryanti)* |  |  |  |  |  |  |  |  |  |  |  |  |  |  | ⇒ |
|  | *Unclassified* |  |  |  |  |  | ⇒ |  |  |  |  |  |  |  |  |  |
|  | *“02d06” (84)* |  |  |  |  |  |  |  |  | ⇒ |  |  |  |  |  |  |
|  | *“SMB53” (85)* |  |  |  |  |  | ⇒ |  |  |  |  |  |  |  |  |  |
|  | *Flavonifractor plautii* |  |  |  |  |  |  |  |  |  | ⇑ |  |  |  |  |  |
|  | *Alkaliphilus* |  |  | ⇓ |  |  |  |  |  |  |  |  |  |  |  |  |
| **Lactobacillales/ *Streptococcaceae*** | *Streptococcus salivarius* | ⇓ | ⇓ |  |  |  |  |  |  |  |  |  |  |  |  |  |
| **Lactobacillales/ *Leuconostocaceae*** | *Weissella* |  |  | ⇓⇓ |  |  |  |  |  |  |  |  |  |  |  |  |
|  | *Leuconostoc* |  |  | ⇓ |  |  |  |  |  |  |  |  |  |  |  |  |
| **Lactobacillales/ *Aerococcaceae*** | *Abiotrophia* |  |  |  | ⇓ | ⇓ |  |  |  |  |  |  |  |  |  |  |
| **Lactobacillales/ *Incertae sedis*** | *Gemella morbillorum* |  |  |  |  |  |  | ⇒ |  |  |  |  |  |  |  |  |
| **Lactobacillales/ *Carnobacteriaceae*** | *Alkalibacterium* |  |  |  | ⇓ | ⇓ |  |  |  |  |  |  |  |  |  |  |
| **Erysipelotrichales/ *Erysipelotrichaceae*** | |  |  |  |  |  |  |  |  |  | ⇑^a^ |  |  |  |  |  |
|  | *Turicibacter sanguinis* | ⇑ | ⇒ |  |  |  |  |  |  |  |  |  |  |  |  |  |
|  | *Catenibacterium* |  |  |  | ⇓ | ⇑ |  |  |  |  |  |  |  |  |  |  |
|  | *Coprobacillus* |  |  |  |  |  | ⇒ |  |  |  |  |  |  |  |  |  |
|  | *Eubacterium* |  |  |  |  |  | ⇒ |  |  |  |  |  |  |  |  |  |
|  | *Holdemania* |  |  |  |  |  |  |  |  | ⇒ |  |  |  |  |  |  |
|  | *Unclassified* |  |  |  |  |  | ⇒ |  |  | ⇒ |  |  |  |  |  |  |
| ***Erysipelotrichales/ Anaeroplasmataceae*** | *Asteroleplasma* |  |  |  |  |  |  |  |  |  |  | ⇑^b^ |  |  |  |  |
| **Tenericutes Phylum** | |  |  |  |  |  |  |  |  |  |  | ⇒ |  |  |  |  |
| **Candidate RF3 Phylum** | |  |  |  |  |  |  |  |  |  |  | ⇒ |  |  |  |  |
| **Tissierelliales/ *Tissierellaceae*** | *Finegoldia* |  |  |  |  |  |  |  |  | ⇒ |  |  |  |  |  |  |
| **Tissierelliales/ *Peptoniphilaceae*** | *Parvimonas* |  |  |  |  |  |  |  |  | ⇒ |  |  |  |  |  |  |
|  | *Anaerovorax* |  |  | ⇓ |  |  |  |  |  |  |  |  |  |  |  |  |
| **Serenomonadales order** | |  |  |  |  |  |  |  |  |  |  |  |  | ⇓⇓ |  |  |
| [***Acidaminococcaceae***](https://en.wikipedia.org/wiki/Acidaminococcaceae) | *Acidaminococcus* |  |  |  |  |  | ⇒ |  |  |  |  |  |  |  |  |  |
| ***Veillonellaceae*** | |  |  |  |  |  |  |  |  |  |  |  |  | ⇓⇓ |  |  |
|  | *Dialister invisus* |  |  | ⇓ |  |  |  |  |  |  |  |  |  |  |  |  |
|  | *Megamonas* |  |  |  |  |  | ⇒ |  |  | ⇒ |  |  |  |  |  |  |
|  | *Megasphaera* |  |  |  |  |  | ⇒ |  |  |  |  |  |  |  |  |  |
|  | *Phascolarctobacterium* |  |  |  |  |  | ⇒ |  |  |  |  |  |  |  |  |  |
|  | *Unclassified* |  |  |  |  |  |  |  | ⇓ |  |  |  |  |  |  |  |
| **Footnote**: Arrows indicate whether the strains are **increased** (⇑), reported as **no significant difference** (⇒), or **decreased** (⇓) in 1) **count** (light grey), 2) **percentage of the total flora** (dark grey), and 3) **variety** (black); single arrow denoting *p*<0.05, double arrows denoting *p* <0.01. ^a^Described in text without definitive *p-*values. ^b^V1V2 datasets are statistically significant, but not V1V3. HC = healthy controls; SIB = sibling without ASD; Autistic Disorder=Severe; Pervasive Developmental Disorder, not otherwise specified (PDD-NOS)=Mild | | | | | | | | | | | | | | | | |

**Additional Table S5.** Changes other bacteria phyla between children with ASD and healthy controls (Single studies)

| **Class/*Family*** | *Genus/ species* | **Adams et al 2011 (26)** | **De Angelis et al 2013 (27)** | | **Finegold et al 2010 (30)** | **Gondalia et al 2010 (32)** | **Gondalia et al 2012(33)** | **Inoue 2016 (34)** | **Kushak et al 2017 (39)** | **Son et al 2015 (44)** | **Strati et al 2017 (46)** | **Williams et al 2011 (50)** | |
| --- | --- | --- | --- | --- | --- | --- | --- | --- | --- | --- | --- | --- | --- |
|  |  |  | Mild vs SIB | Severe vs SIB |  | ASD vs HC | ASD vs SIB |  |  |  |  | Ileal | Cecal |
| ***Gammaproteobacteria class*** | |  |  |  |  |  |  |  |  |  |  | ⇒ | ⇒ |
| ***Enterobacteriaceae*** | *Citrobacter* |  |  |  |  | ⇒ |  |  |  |  |  |  |  |
|  | *C. youngae* | ⇒ |  |  |  |  |  |  |  |  |  |  |  |
|  | *C. braakii* | ⇒ |  |  |  |  |  |  |  |  |  |  |  |
|  | *C. freundii* | ⇒ |  |  |  |  |  |  |  |  |  |  |  |
|  | *Proteus mirabilis* | ⇒ |  |  |  |  |  |  |  |  |  |  |  |
|  | *Enterobacter cloacae* | ⇒ |  |  |  |  |  |  |  |  |  |  |  |
|  | *Klebsiella oxytoca* | ⇓ |  |  |  |  |  |  |  |  |  |  |  |
|  | *K. pneumonia* | ⇒ |  |  |  |  |  |  |  |  |  |  |  |
|  | *Salmonella* | ⇒ |  |  |  |  |  |  |  |  |  |  |  |
|  | *Pseudomonas aeruginosa* | ⇒ |  |  |  |  |  |  |  |  |  |  |  |
|  | *Others* |  |  |  |  |  |  | ⇒ |  |  |  |  |  |
| **Alphaproteobacteria class** | |  |  |  |  |  |  |  |  |  |  | ⇒ | ⇒ |
| ***Anaplasmataceae*** | *Wolbachia* |  |  |  |  |  | ⇓ |  |  |  |  |  |  |
| ***Hyphomicrobiaceae*** | *Devosia* |  |  |  |  |  |  |  | ⇓ |  |  |  |  |
| ***Thalassospira*** |  |  |  |  |  |  |  |  |  | ⇑^b^ |  |  |  |
| **Betaproteobacteria class** | |  |  |  |  |  |  |  |  |  |  | ⇒ | ⇑ |
| ***Alcaligenaceae*** | |  |  |  |  |  |  | ⇒ |  |  |  | ⇑^a^ | ⇑^a^ |
| ***Sutterellaceae*** | *Parasutterella excrementihominis* |  | ⇒ | ⇑ |  |  |  |  |  |  |  |  |  |
| ***Burkholderiaceae*** | *Burkholderia* |  |  |  |  |  |  |  | ⇑ | ⇑^b^ |  |  |  |
|  | *Ralstonia* |  |  |  |  |  |  |  | ⇑ |  |  |  |  |
| ***Comamonadaceae*** | |  |  |  |  |  |  |  |  | ⇑^c^ |  |  |  |
| ***Neisseriaceae*** | *Neisseria* |  |  |  |  |  |  |  | ⇓ |  |  |  |  |
| **Deletaproteobacteria class** | |  |  |  |  |  |  |  |  |  |  | ⇒ | ⇒ |
| ***Desulfovibrionaceae*** | *Bilophila* |  |  |  |  |  |  | ⇒ |  |  | ⇓⇓ |  |  |
|  | *Desulfovibrio* |  |  |  | ⇑ |  |  | ⇒ |  |  |  |  |  |
|  | *D. desulfuricans* |  |  |  | ⇑ |  |  |  |  |  |  |  |  |
|  | *D. intestinalis* |  |  |  | ⇑ |  |  |  |  |  |  |  |  |
|  | *D. piger* |  |  |  | ⇑ |  |  |  |  |  |  |  |  |
| **Epsilonproteobacteria class** | |  |  |  |  |  |  |  |  |  |  | ⇒ | ⇒ |
| **Tenericutes phylum** | |  |  |  | ⇒ |  |  |  |  |  |  |  |  |
| **Cyanobacteria phylum** | |  |  |  | ⇒ |  |  |  |  | ⇒ |  |  |  |
| **Chroococcales** | *Chloroplast* |  |  |  |  |  |  |  |  | ⇑ |  |  |  |
| ***Bifidobacteriaceae*** | *Bifidobacterium angulatum* |  |  |  | ⇒ |  |  |  |  |  |  |  |  |
|  | *B. animalis* |  |  |  | ⇒ |  |  |  |  |  |  |  |  |
|  | *B. bifidum* |  |  |  | ⇒ |  |  |  |  |  |  |  |  |
|  | *B. catenulatum* |  | ⇑ | ⇒ |  |  |  |  |  |  |  |  |  |
|  | *B. dentium* |  |  |  | ⇒ |  |  |  |  |  |  |  |  |
|  | *B. longum* |  |  |  | ⇓ |  |  |  |  |  |  |  |  |
|  | *B. pseudocatenulatum* |  |  |  | ⇒ |  |  |  |  |  |  |  |  |
|  | *B. pseudolongum* |  |  |  | ⇒ |  |  |  |  |  |  |  |  |
|  | *B. saeculare (Bifidobacterium pullorum* subsp. *saeculare)* |  |  |  | ⇒ |  |  |  |  |  |  |  |  |
| **Footnote**: Arrows indicate whether the strains are **increased** (⇑), reported as **no significant difference** (⇒), or **decreased** (⇓) in 1) **count** (light grey), 2) **percentage of the total flora** (dark grey), and 3) **variety** (black); single arrow denoting *p*<0.05, double arrows denoting *p* <0.01. ^a^Described in text without definitive *p-*values. ^b^V1V2 datasets are statistically significant, but not V1V3. ^c^V1V3 datasets are  statistically significant, but not V1V2. HC = healthy controls; SIB = sibling without ASD; Autistic Disorder=Severe; Pervasive Developmental Disorder, not otherwise specified (PDD-NOS)=Mild | | | | | | | | | | | | | |
